# Determinants of Healthcare‐Seeking Preferences of Female Sex Workers: A Cross‐Sectional Study From the South‐Western Region of Bangladesh

**DOI:** 10.1002/hsr2.72592

**Published:** 2026-06-04

**Authors:** Shaharior Rahman Razu, Kim Usher, Rikki Jones, Md. Shahidul Islam

**Affiliations:** ^1^ School of Health University of New England New England New South Wales Australia; ^2^ Sociology Discipline Khulna University Khulna Bangladesh

**Keywords:** Bangladesh, determinants, female sex workers, government hospitals, healthcare choice, NGO

## Abstract

**Background and Aims:**

Female sex workers face substantial barriers to accessing healthcare services. We examined factors influencing preference for government versus non‐government healthcare services among female sex workers in Bangladesh.

**Methods:**

Three hundred and eighty‐four female sex workers from the south‐western region of Bangladesh were interviewed in this cross‐sectional survey. Preferences and explanatory variables (demographics, income, abuse history, routine check‐up engagement, etc.) were analyzed using *χ* ^2^ tests and multiple logistic regression (MLR) to identify independent predictors of government‐facility preference.

**Results:**

We recorded that about one‐third (32.1%) of our participants preferred government services. Participants reporting a routine health check‐up had over twice the odds of government‐care preference (AOR = 2.72, 95% CI 1.41–5.24), whereas non‐Muslims (AOR = 0.21, 95% CI 0.07–0.67) and those experiencing abuse (AOR = 0.35, 95% CI 0.16–0.73) were less likely to choose public facilities. Higher incomes decreased the likelihood of choosing government facilities (AOR = 0.23–0.30), while moderate household size (3–4 members; AOR = 2.41, 95% CI 1.11–5.23) and urban residence (AOR = 2.41, 95% CI 0.99–5.87) increased it.

**Conclusion:**

We conclude that engagement with routine services, socioeconomic status, religion, and abuse experiences shaped the healthcare‐seeking preferences for the female sex workers. Tailored interventions, including stigma‐reduction training, outreach to minority groups, and NGO–public partnerships, are needed to improve government‐facility utilization among this marginalized social group in Bangladesh.

## 1 Introduction

Female sex workers (FSWs) in Bangladesh operate within a challenging healthcare landscape. This challenge is characterized by a certain degree of legal ambiguity, social stigma, and limited resources, which collectively negatively affect their accessibility to healthcare services. Although sex work itself is not fully criminalized in Bangladesh, its operation remains governed through court directives, municipal regulations, policing practices, and informal controls, which creates an uncertainty in everyday life for many female sex workers. In practice, this means that these women may face identity verification demands, eviction threats, periodic raids, harassment, or pressure from local power structures, which affects routine contact with formal institutions, including health services. Although the Upazila Health Complexes and district hospitals serve as basic elements for the country's public health system, lack of doctors and nurses, inadequate medical drugs and equipment, and the judgmental characteristics of healthcare providers often deter female sex workers from seeking care at these government facilities, despite the relatively low fees [1]. Furthermore, the bureaucratic complexities in the legal and administrative system of the government and restricted operating hours of the public system exacerbate these obstacles, making health check‐ups for conditions such as cervical cancer screening, HIV, and STI testing inconsistent but deemed essential for the female sex workers [2, 3]. For example, the absence of official identification documents, fear of disclosing occupation, and concerns about confidentiality during registration procedures may delay or prevent attendance at public healthcare facilities.

The non‐governmental entities, including large NGOs such as BRAC and Marie Stopes, as well as smaller drop‐in centers, have developed specialized services that ensure confidentiality, offer flexible scheduling, and engage in targeted outreach. These entities often utilize female sex workers as outreach workers to build trust and guarantee the provision of services such as condom distribution, STI screening, and basic counseling in a nonjudgmental environment offering more flexibility [4, 5]. However, the relative convenience and privacy of non‐government facilities come with some limitations, as the service costs may be unaffordable for lower‐income workers, and the services provided by these clinics frequently lack comprehensive medical diagnostics or long‐term management of chronic health conditions [6, 7].

The existing literature highlights various socio‐demographic and occupational factors that influence the healthcare choices of female sex workers. Female sex workers of younger age, typically possessing higher levels of education and health literacy, are more inclined to utilize formal services, both public and private, while older and residential‐based female sex workers often depend on informal or traditional practitioners due to barriers such as cost, distance, or stigma that hinder access to mainstream providers [1, 8]. The income level of female sex workers has a paradoxical effect on their healthcare. While higher earnings facilitate access to private care, they are often associated with increased client loads, resulting in limited time for routine check‐ups. In contrast, individuals with limited disposable income frequently postpone seeking medical care until acute symptoms require them to visit a hospital, at which stage conditions like untreated hypertension or advanced STIs may already have resulted in serious complications [9, 10].

Behavioral determinants, including previous experiences of violence and abuse, harassment, and discrimination, significantly influence decisions regarding care‐seeking. Female sex workers who experience negative interactions, including social stigma, dismissive treatment by nurses, and outright service refusal, are more inclined to avoid government facilities in favor of NGO‐operated clinics, which they consider safer, although more expensive [8, 11]. Geographic accessibility adds an additional layer of susceptibility for healthcare‐related matters at the district levels of Bangladesh [12]. The closeness of public hospitals and private clinics facilitates a more equitable distribution of service utilization, whereas the remote areas typically have only inadequately equipped rural health centers as public facilities [13], and NGOs often lack the necessary outreach capacity although these areas are often locations for brothel‐based or street‐based sex work. These geographic disparities are particularly relevant in remote areas where transport costs, travel time, and social visibility during travel may further discourage women from seeking preventive care.

Despite these issues, limited research has directly compared the utilization of government and non‐government facilities among female sex workers in Bangladesh. The present study focuses on the south‐western region because this area represents an important yet comparatively underexplored setting of sex work in Bangladesh. This particular area contains border districts, transport corridors, riverine routes, urban municipalities, and rural settlements where sex work occurs in diverse forms, including street‐based, residence‐based, and hidden networks. The region also includes areas with varying levels of NGO presence and uneven public health infrastructure, making it a useful context for examining how service environment shapes healthcare choices. Compared with Dhaka and a few other studied locations, evidence from south‐western Bangladesh remains limited despite the presence of socially and economically vulnerable populations. This study attempts to address the existing gap by surveying the female sex workers in the south‐western region of the country, documenting their self‐reported preferences and examining the factors that influence these choices. We aim to model the preference as a binary outcome (government facility vs. non‐government healthcare facility) while adjusting for key covariates to quantify the relative influence of factors including personal characteristics, family structure, income and expenditure, occupational setting, and experiences of abuse. Understanding these determinants is important because the type of facility chosen can influence continuity of care, timeliness of treatment, preventive screening uptake, and the financial burden for this vulnerable group of women. By identifying which subgroups are excluded from particular services, the findings can help direct scarce resources toward more equitable and responsive healthcare delivery. The study offers practical recommendations for policymakers and strategy planners to improve the sensitivity training and supply management in public hospitals, as well as subsidize the NGO services for the marginalized female sex workers.

## 2 Methods and Materials

### 2.1 Study Design and Context

This study utilized a cross‐sectional quantitative design to investigate the factors influencing healthcare choices among female sex workers from Khulna, Satkhira, and Jessore districts located in the southwestern region of Bangladesh. The selection of these districts is based on their established sex work activity and our prior collaborations with local organizations. The survey research design was chosen for its well‐established effectiveness in documenting contemporary behavioral trends and guiding potential policy interventions aimed at marginalized groups [14, 15].

### 2.2 Participants and Sampling Strategy

A total of 384 female sex workers, aged 18 and older, were surveyed during the data collection period. We included only cisgender women in this study; therefore, no transgender women or members of the hijra community were considered. The participants were involved in sex work during the data collection time as per the selection criteria. Given the social stigmatization, concealed nature, and the ambiguous legal status of sex work within the Bangladeshi context, a purposive sampling technique was utilized to access the hard‐to‐reach female sex workers. This method facilitated participant engagement via established networks that included outreach workers, peer educators, and community‐based NGOs.

The sample size was calculated using the following Cochran's formula to ensure statistical reliability at a 95% confidence level:

$$n=\frac{z^{2}\times p(1-p)}{d^{2}}\;,$$

Where

*Z* = 1.96 (for 95% confidence level).

*p *= assumed proportion for maximum variance.

*d *= Allowable maximum error in estimating population proportion (5% significance level).

The result obtained using this formula yields a substantial sample size of 384, enabling for generalizable conclusions with a 95% confidence level, while balancing feasibility with the necessity for sufficient diversity and the logistical requirements for robust data. Participants were recruited through an advertisement via NGO networks located in the urban centers of the chosen districts, ensuring adherence to ethical and methodological standards. We also used our previous connections and referrals to reach the potential participants, apart from that. The participants' eligibility was established through a thorough analysis interview of the criteria, such as age (18 years and above), current engagement in sex work, residency in designated districts, and informed consent.

### 2.3 Variables

The dependent variable in this study was healthcare preference, which we categorized as the respondent's typical choice between government and non‐government healthcare services when seeking medical care. Responses were classified into “government” (referring to public facilities), and “non‐government” categories (referring to private clinics, NGO services, pharmacies, or traditional providers). The independent variables included various sociodemographic and behavioral factors, as guided by prior research. Age categories were defined as 18–25, 26–35, and 36 years and above; districts were classified as Khulna, Satkhira, and Jessore; religion was categorized into Muslim and non‐Muslim; and residence was categorized as rural or urban. Household characteristics comprised the number of family members (1–2, 3–4, 5 and above) and family structure (nuclear or joint).

Economic variables included monthly income categories (BDT 6000–10,000; 10,001–15,000; 15,001–20,000; 20,001 and above) and healthcare expenditure categories (BDT 200–500, 501–1000, 1001–1500, 1501 and above). Occupational details included the type of sex work (brothel/hotel‐based, residential‐based, street‐based) and the regularity of engagement (part‐time or full‐time). Additional factors considered were age at entry into sex work (12–15, 16–20, 21–25, 26 and above), daily client count (2, 3, 4, 5, and above), and history of abuse (yes/no). Routine health check‐up behavior, characterized by participation in screenings every 3–6 months, was also incorporated as an independent variable.

### 2.4 Data Collection

Data were collected using a structured questionnaire administered by trained field researchers with backgrounds in social sciences and experience of working with vulnerable social groups. The survey with a researcher‐led questionnaire was often conducted in mutually convenient locations, including NGO offices that work closely with the female sex workers. Interviews were also conducted at participants' preferred locations, such as their homes or workplaces prioritizing their comfort and confidentiality. We advertised for survey participants through the local organizations of our past connections in the research area. This alliance allowed female sex workers who were afraid of stigma or legal implications to participate in this study. Participants were selected following a brief screening process to verify they met the inclusion criteria of this research.

The questionnaire encompassed items related to sociodemographic characteristics and healthcare‐seeking behaviors, as it was formulated following a comprehensive literature review [10, 16, 17, 18, 19, 20, 21, 22]. The instrument underwent pre‐testing with a small cohort of sex workers, who were not part of the main sample, to enhance the clarity, coherence, and sensitivity of the items. Modifications were implemented as necessary. We collected the data from February 2023 to April 2023 through face‐to‐face interviews.

### 2.5 Data Analysis

The data analysis was conducted utilizing SPSS software (version 29), and descriptive statistics were employed to delineate participant characteristics. The examinations of associations between healthcare preferences and independent variables were conducted using chi‐square tests for categorical data first. Binary logistic regression was employed to identify predictors of government healthcare preference in the next step. Results were presented as odds ratios (OR) accompanied by 95% confidence intervals (CI) showing the statistically significant associations between variables. This technique facilitated a detailed comprehension of the various factors that affected healthcare‐seeking preferences within the marginalized female sex workers.

### 2.6 Ethical Approval

Approval for ethical considerations was granted from the Human Research Ethics Committee (HREC) at the University of New England, Australia (HE22‐199). All participants were provided an information sheet outlining the purpose of this research, the procedure, and the voluntary nature of their involvement. Informed consent was obtained prior to participation, with information sheets available in both English and Bangla to address literacy requirements. Participants were guaranteed total confidentiality and were assured that no identifiable personal information was gathered. Anonymity was preserved during data management and reporting, consistent with the approved research protocol.

## 3 Results

Our findings show that healthcare preferences among female sex workers were shaped by intersecting factors such as place of residence, religion, income, type of sex work, age of entry, and engagement with routine health services (Table 1). Among the 384 female sex workers surveyed, about one‐third of the participants (32.1%) preferred government healthcare facilities, while the remaining67.9% sought care from non‐government sources. Preferences varied notably by several demographic and occupational characteristics, as urban residents (92.7%) were more likely to use government services than rural participants (7.3%), and Muslim respondents (96.7%) showed a strong preference for government facilities, while 91.7% of non‐Muslims opted for non‐governmental options in this study.

**Table 1:** Distribution of variables of the respondents by healthcare‐seeking preferences.

Variables	*n* (%)	Healthcare‐seeking preferences		*χ* ^2^ value	*p* value
		Government	Non‐govt.		
Overall	384	*n* (%)	*n* (%)		
Age				3.36	0.505
18–25	52 (13.6)	13 (10.6)	39 (14.9)		
26–35	196 (51.0)	65 (52.8)	131 (50.2)		
36 and above	136 (35.4)	45 (36.6)	91 (34.9)		
District				0.72	0.698
Khulna	261 (67.9)	80 (65.1)	181 (69.3)		
Satkhira	75 (19.6)	26 (21.8)	49 (18.8)		
Jessore	48 (12.5)	17 (13.1)	31 (11.9)		
Religion				14.15	< 0.001
Muslim	336 (87.5)	119 (96.7)	217 (83.1)		
Non‐muslim	48 (12.5)	04 (3.3)	44 (16.9)		
Residence				8.11	0.004
Rural	57 (14.8)	9 (7.3)	48 (18.4)		
Urban	327 (85.2)	114 (92.7)	213 (81.6)		
Number of family members				0.84	0.662
1–2	91 (23.7)	26 (21.1)	65 (24.9)		
3–4	186 (48.4)	60 (48.8)	123 (48.3)		
5 and above	107 (27.9)	37 (30.1)	70 (26.8)		
Type of family				3.23	0.071
Nuclear	279 (72.7)	82 (66.7)	197 (75.5)		
Joint	105 (27.3)	41 (33.3)	64 (24.5)		
Monthly Income				16.67	< 0.001
6000–10,000	70 (18.2)	32 (26.0)	38 (14.6)		
10,001–15,000	120 (31.3)	47 (38.2)	73 (28.0)		
15,001–20,000	138 (35.9)	32 (26.0)	106 (40.6)		
20,001 and above	56 (14.6)	12 (9.8)	44 (16.9)		
Monthly expenditure on healthcare				1.076	0.623
200–500	122 (31.8)	43 (35.0)	79 (30.3)		
501–1000	117 (30.5)	33 (26.8)	84 (32.2)		
1001–1500	29 (7.6)	8 (6.5)	21 (8.0)		
1501 and above	116 (30.2)	39 (31.7)	77 (29.5)		
Type of sex worker				8.46	0.015
Brothel/hotel‐based	72 (18.8)	14 (11.4)	58 (22.2)		
Residential‐based	131 (34.1)	40 (32.5)	91 (34.9)		
Street‐based	181 (47.1)	69 (56.1)	112 (42.9)		
Regularity in sex work				0.26	0.613
Full‐time	371 (96.6)	118 (95.9)	253 (96.9)		
Part‐time	13 (3.4)	5 (4.1)	8 (3.1)		
Age of starting sex work				8.15	0.043
12–15	46 (11.9)	15 (12.2)	31 (11.9)		
16–20	201 (52.3)	56 (45.5)	145 (55.6)		
21–25	100 (26.1)	43 (35.0)	57 (21.8)		
26 and above	37 (9.7)	9 (7.3)	28 (10.7)		
Number of clients served per day				6.52	0.089
2	84 (21.9)	23 (18.7)	61 (23.4)		
3	132 (34.4)	51 (41.5)	81 (31.0)		
4	108 (28.1)	27 (22.0)	81 (31.0)		
5 and above	60 (15.7)	22 (17.9)	38 (14.6)		
Abused physical/verbally				10.07	0.002
No	311 (80.9)	112 (90.2)	200 (76.6)		
Yes	73 (19.1)	12 (9.8)	61 (23.4)		
Routine health check‐up				13.64	< 0.001
Yes	246 (64.1)	95 (77.2)	28 (22.8)		
No	138 (35.9)	151 (57.9)	110 (42.1)		

We found that income influenced preferences in healthcare choices, with 26.0% of those earning BDT 6000–10,000 and 38.2% of those earning BDT 10,001–15,000 favoring government services. In contrast, 40.6% of those earning BDT 15,001–20,000 and 16.9% of those above BDT 20,000 opted for non‐government providers. Street‐based sex workers reported the highest government healthcare usage (56.1%), compared to residential‐based (32.5%) and brothel/hotel‐based workers (11.4%). Those working full‐time (95.9%) were more likely to use government services than the part‐timers (4.1%). Participants who began sex work between the ages of 21–25 years showed higher use of government facilities (35.0%), while those starting earlier leaned more towards non‐government options.

Female sex workers who served three clients per day had the highest proportion using government services (41.5%), while those with two (18.7%) or five or more clients (17.9%) leaned towards non‐governmental care. Importantly, 77.2% of those who had a routine health check‐up in the past 6 months used government healthcare, compared to only 42.1% among those who did not. Table 1 also presents the association between healthcare‐seeking preferences and various explanatory variables using a *χ* ^2^ test of independence. The results show that religion (*p* < 0.001), residence (*p* = 0.004), monthly income (*p* < 0.001), type of sex worker (*p* = 0.015), age at starting sex work (*p* = 0.043), abused physically/verbally (*p* = 0.002), and routine health check‐up (*p* < 0.001) were significantly associated with the healthcare‐seeking preferences of the female sex workers.

Several factors were significantly associated with female sex workers' preference for government healthcare services in our multivariable analysis (Table 2). We found that those undergoing routine check‐ups had over twice the odds of preferring government care (AOR = 2.72, 95% CI 1.41–5.24). Non‐Muslim participants were less likely to prefer government options than Muslims (AOR = 0.21, 95% CI 0.07–0.67), and experience of abuse was linked to lower odds of government healthcare preference (AOR = 0.35, 95% CI 0.16–0.73). On the other hand, higher income showed an inverse relationship as those earning BDT 15,001–20,000 (AOR = 0.30, 95% CI 0.12–0.78) and above 20,000 (AOR = 0.23, 95% CI 0.07–0.73) had a lower probability compared to those earning BDT 6000–10,000.

**Table 2:** Multiple logistic regression of variables associated with healthcare‐seeking preferences.

Variables	Coefficient *β*	Odds ratio, exp(*B*)	95.0% CI		*p* value
			Lower	Upper	
Age					
18–25*		1.00			0.840
26–35	−0.275	0.759	0.303	1.900	0.556
36 and above	−0.257	0.773	0.289	2.068	0.609
District					
Khulna*		1.00			0.823
Satkhira	0.225	1.252	0.617	2.540	0.534
Jessore	0.106	1.112	0.473	2.613	0.808
Religion					
Muslim*		1.00			
Non‐muslim	−1.572	0.208	0.065	0.668	0.008
Residence					
Rural*		1.00			
Urban	0.877	2.405	0.985	5.872	0.054
Number of family members					
1–2*		1.00			0.062
3–4	0.879	2.407	1.107	5.234	0.027
5 and above	0.468	1.596	0.560	4.548	0.381
Type of family					
Nuclear*		1.00			
Joint	0.730	2.074	0.938	4.589	0.072
Monthly income					
6000–10,000*		1.00			0.018
10,001–15,000	−0.417	0.659	0.285	1.523	0.329
15,001–20,000	−1.205	0.300	0.115	0.779	0.013
20,001 and above	−1.465	0.231	0.074	0.727	0.012
Monthly expenditure on healthcare					
200–500*		1.00			0.518
501–1000	−0.254	0.776	0.388	1.554	0.474
1001–1500	0.313	1.368	0.464	4.033	0.570
1501 and above	0.288	1.333	0.597	2.978	0.483
Types of sex workers					
Brothel/hotel‐based*		1.00			0.340
Residential‐based	0.409	1.506	0.584	3.881	0.397
Street‐based	0.703	2.020	0.790	5.162	0.142
Regularity in sex work					
Full‐time*		1.00			
Part‐time	0.156	1.169	0.280	4.884	0.830
Age of starting sex work					
12–15*		1.00			0.025
16–20	−0.013	0.987	0.433	2.250	0.975
21–25	1.026	2.791	1.068	7.292	0.036
26 and above	0.393	1.481	0.434	5.054	0.530
Number of clients served per day					
2*		1.00			0.228
3	0.654	1.924	0.945	3.919	0.071
4	0.179	1.196	0.551	2.600	0.651
5 and above	0.649	1.914	0.704	5.203	0.203
Abused physical/verbally					
No*		1.00			
Yes	−1.063	0.346	0.164	0.729	0.005
Routine health check‐up					
No*		1.00			
Yes	0.999	2.717	1.410	5.236	0.003

* Reference category.

Participants who were from a family size of 3–4 members were more likely to prefer government services (AOR = 2.41, 95% CI 1.11–5.23), while respondents from joint families showed a non‐significant probability for the same option (AOR = 2.07, 95% CI 0.94–4.59, *p *= 0.072). Female sex workers from urban areas had marginally higher odds (AOR = 2.41, 95% CI 0.99–5.87, *p *= 0.054), and those who began sex work between ages 21–25 were more likely to prefer public healthcare than those starting at 12–15 (AOR = 2.79, 95% CI 1.07–7.29). The rest of the variables, including age, district, healthcare spending, type and frequency of sex work, and client volume, were not found to be significantly associated.

## 4 Discussion

Findings from our multivariate analysis show that engagement with routine health check‐ups, religious affiliation, experiences of abuse, income level, household composition, place of residence, and age at entry into sex work each exerted significant influences on service preferences of the female sex workers in Bangladesh. Women who had undergone a routine health check‐up in the preceding 6 months were twice as likely to prefer government facilities compared to those who had not. This finding aligns with the existing literature, which argues that previous correspondence with formal health systems may have played a role in building trust and familiarity, leading to a positive perception of service quality and affordability, and encouraging continued use of public services [1]. We assume that routine engagement can reduce stigma and administrative procedure‐related doubts through positive interactions with hospital staff and the setting. This may have created opportunities for tailored health education, which can reinforce a government service preference [1, 23].

Religion emerged as a strong determinant, as non‐Muslim participants exhibited significantly lower odds of preferring government care than their Muslim counterparts. Evidence from nationally representative surveys from Bangladesh also reflects the difference in community norms and levels of comfort with public facilities [24]. Minority religious groups often fear discrimination and cultural insensitivity, prompting them to seek care in non‐governmental clinics often run by faith‐based or NGO providers that offer targeted support for these groups [25]. Conversely, the stronger preference for government facilities among Muslim participants may also reflect their position as the demographic majority within Bangladesh, where public institutions largely operate within social and cultural norms familiar to Muslim communities. Shared language practices, religious observances, gender expectations, dress norms, and holiday calendars may create a greater sense of belonging and procedural comfort when navigating government services. In this context, Muslim participants may anticipate fewer identity‐based barriers or misunderstandings, which can increase confidence in seeking care from public providers. Additionally, female sex workers who experienced any form of physical or verbal abuse were less likely to visit government care services. We assume that the victims of violence may be afraid of judgment and privacy concerns, or may have experienced negative encounters when accessing public facilities [26, 27]. NGOs and private clinics, on the other hand, often perceived as more discreet and trauma‐ responsive and gender sensitive tailored care for vulnerable social groups [28, 29].

Economic factors also played a crucial role in determining the healthcare choices among the participants. Compared to the lowest income group (BDT 6000–10,000), those earning BDT 15,001–20,000 and above BDT 20,000 had a substantially lower likelihood of preferring government services. Previous studies also reported that higher‐earning sex workers may perceive non‐governmental providers as offering higher quality and shorter wait times in addition to perceived increased privacy [1, 30]. Conversely, lower‐income workers likely rely on the subsidized or free care available at government facilities to meet their routine and emergent healthcare needs, as they are unwilling to spend a significant out‐of‐pocket fee [30, 31]. We observed that those from moderate‐sized families of 3–4 members were more than twice as likely to choose government services compared to smaller households (1–2 members). This possibly happened due to economic reasons again, as larger families may benefit from the cost savings of public care, whereas very small or very large (joint) family structures may either lack sufficient financial incentive or experience complex decision‐making dynamics around healthcare expenditure, which is often deemed as a secondary priority [32].

Higher odds of preferring public facilities were observed among urban residents and those who began sex work between the ages of 21 and 25. Urban dwellers may have greater proximity and awareness of well‐equipped district‐level government hospitals [13], while we argue that the mid‐career entrants into sex work might retain prior habits of using public healthcare from before entering the profession. Overall, these findings underscore the importance of enhancing the responsiveness and inclusivity of government healthcare services for female sex workers in Bangladesh. Targeted interventions and strategies, including trauma‐responsive staff training, culturally sensitive outreach to religious minorities, and flexible services that accommodate vulnerable income and family groups. Given the findings, aligning service delivery with the lived realities of female sex workers, policymakers, and health planners can promote more equitable access and better health outcomes for this underserved group.

Findings of our study indicate that the preference of many female sex workers for NGO and private clinics underscores the necessity for government services to adopt a more trauma‐informed, culturally sensitive, and accessible approach. Training healthcare personnel in nonjudgmental and confidential care has the potential to diminish stigma and restore trust [33, 34]. Additionally, outreach efforts targeting minority and faith‐based groups may enhance acceptability. Implementing practical adaptations, including private consultation spaces, flexible hours, and mobile clinics, would reduce barriers for women with complex schedules or residing in remote areas [35, 36]. Financial support mechanisms, such as subsidized care or vouchers, can mitigate cost‐related exclusion [37]. Formal referral pathways between NGOs and government facilities, in conjunction with peer navigators from FSW communities, can enhance continuity and promote uptake [38]. Beyond service‐level reforms, the broader legal and policy environment also needs further attention. Growing international evidence suggests that criminalized or legally ambiguous contexts may discourage disclosure, increase fear of harassment, and reduce timely healthcare seeking among sex workers. WHO and UNAIDS have increasingly recognized that rights‐based approaches, including movement toward decriminalization and legal protections, can support safer working conditions and better engagement with health services. Recent evidence syntheses further indicate that decriminalization is associated with improved access to prevention, treatment, and social protection services for sex workers [39]. While legal reform was beyond the scope of the present study, it remains an important structural consideration for improving long‐term health equity in Bangladesh. Aligning service delivery with identified needs and monitoring patient experiences enables policymakers to enhance equitable access and improve health outcomes for this underserved population.

Although purposive sampling was effective in reaching the frequently elusive population of female sex workers, the findings should be regarded with caution concerning broader generalizability. Recruitment was conducted via established relationships with local non‐governmental and community‐based organizations in the southwestern region of Bangladesh. Consequently, those who are less involved with these networks or reside in more isolated regions may have been underrepresented, despite encountering significant obstacles to healthcare access. To address this, we partnered with various organizations and incorporated participants from four different subgroups of female sex workers to ensure representation throughout the population. We also acknowledge that this study considered only cisgender women, which may have limited its scope.

A further limitation is the dependence on memory calls in survey data, which may be influenced by recollection inaccuracies and social desirability bias. Due to the stigma and legal complexities associated with sex work, participants may have either under‐reported or over‐reported their healthcare utilization. We sought to mitigate this by prioritizing anonymity, enforcing stringent secrecy, and establishing a secure atmosphere to promote candid responses. The cross‐sectional design limits our capacity to monitor temporal changes in health‐seeking behavior. Future longitudinal research will yield significant insights into the changing patterns and trends among female sex workers.

## 5 Conclusion

This paper explores the determinants influencing healthcare service preference (government vs. non‐government) among female sex workers in Bangladesh. We identified that a history of routine check‐ups, Muslim religious affiliation, lower income, moderate household size, urban residence, and later entry into sex work were associated with a higher likelihood of opting for government facilities, while experiences of abuse and higher earnings tend to shift preferences toward non‐government options. The findings underscore the need to enhance government facilities through trauma‐informed training, culturally sensitive outreach, and streamlined services, while promoting NGO‐public partnerships to exchange best practices in confidentiality and patient engagement. Tailored interventions addressing the socioeconomic and experiential realities of sex workers are essential for expanding equitable access and improving health outcomes among vulnerable female sex workers in Bangladesh.

## Author Contributions

**Shaharior Rahman Razu:** conceptualization, investigation, funding acquisition, writing – original draft, methodology, validation, visualization, project administration, formal analysis, software, data curation, and resources. **Kim Usher:** conceptualization, methodology, validation, visualization, writing – review and editing, data curation, and supervision. **Rikki Jones:** conceptualization, methodology, validation, visualization, writing – review and editing, data curation, and supervision. **Md Shahidul Islam:** conceptualization, methodology, validation, visualization, writing – review and editing, data curation, and supervision.

## Conflicts of Interest

The authors declare no conflicts of interest.

## Transparency Statement

The lead author, Shaharior Rahman Razu, affirms that this manuscript is an honest, accurate, and transparent account of the study reported; that no important aspects of the study have been omitted; and that any discrepancies from the study as planned (and, if relevant, registered) have been explained.

## Data Availability

The data that support the findings of this study are available on request from the corresponding author. The data are not publicly available due to privacy or ethical restrictions.
